# The density of bone marrow mononuclear cells and CD34+ cells in patients with three neurologic conditions

**DOI:** 10.1186/s12883-023-03071-3

**Published:** 2023-01-23

**Authors:** Kien Trung Nguyen, Nhung Thi My Hoang, Hoang-Phuong Nguyen, Liem Nguyen Thanh

**Affiliations:** 1 Vinmec Research Institute of Stem Cell and Gene Technology, Vinmec Healthcare System, 458 Minh Khai, Hanoi, Vietnam; 2 University of Science, Vietnam National University, 334 Nguyen Trai, Hanoi, Vietnam; 3 College of Health Science, VinUniversity, Vinhomes Ocean Park, Gia Lam District, Hanoi, Vietnam

**Keywords:** Bone marrow, Mononuclear cells, CD34+ cells, Cerebral palsy, Traumatic brain injury

## Abstract

**Background:**

This study aimed to identify the density of mononuclear cells (MNCs) and CD34+ cells in the bone marrow of patients with three neurologic conditions.

**Methods:**

The study included 88 patients with three neurologic conditions: 40 with cerebral palsy (CP) due to oxygen deprivation (OD), 23 with CP related to neonatal icterus (NI), and 25 with neurological sequelae after traumatic brain injury. Bone marrow aspiration was conducted from the patients’ bilateral anterior iliac crest under general anesthesia in an operating theater. MNCs were isolated by Ficoll gradient centrifugation and then infused intrathecally.

**Results:**

There was a significant difference in the average MNC per ml and percentage of CD34+ cells by the type of disease, age group, and infusion time (*p* value < 0.05). The multivariable regression model showed the percentage of CD34+ association with the outcome (gross motor function 88 items- GMFM-88) in patients with CP.

**Conclusions:**

The density of MNCs was 5.22 million cells per mL and 5.03% CD34+ cells in patients with three neurologic conditions. The highest density of MNCs in each ml of bone marrow was found in patients with CP due to OD, whereas the percentage of CD34+ cells was the highest among patients with CP related to NI.

**Supplementary Information:**

The online version contains supplementary material available at 10.1186/s12883-023-03071-3.

## Background

Bone marrow mononuclear cells (BMMNCs) consist of hemopoietic stem cells and mesenchymal stem cells together with progenitors of endothelial cells or somatic cells [1, 2]. These cells produce cytokines or immunomodulatory and neurotrophic factors that can contribute to central nervous system repair, neurogenesis, neuroregeneration, and neuroreplacement [1–6].

Mononuclear cell (MNC) infusion has been shown to be safe and efficacious in different neurologic conditions, such as cerebral palsy and neurological sequelae after traumatic brain injury [7–10].

Cerebral palsy (CP) comprises a heterogeneous group of neurological disorders and is one of the most common physical disabilities observed in infants, with a prevalence of approximately two children in 1000. Different antenatal, perinatal, and postneonatal factors are responsible for CP, which results in defects or lesions in the immature brain [11]. Hypoxia and neonatal icterus are the most common causes of CP in developing countries.

Traumatic brain injury (TBI) produces both acute and chronic consequences that lead to permanent disabilities that increase long-term mortality and reduce life expectancy [12]. The majority of survivors of moderate and severe TBI have chronic neurobehavioral sequelae, including cognitive deficits, cognitive motor deficits, changes in personality and increased rates of psychiatric illness [12, 13]. The rate of sequelae after traumatic brain injury in the community is 17% [14, 15].

Although clinical trials have shown the safety and efficiency of BMMNC infusion for CP [10, 13–17] and neurological sequelae after TBI [8, 18–21], no studies have compared the correlation of the density of mononuclear cells (MNCs) and the percentage of CD34+ cells with different neurologic conditions, including cerebral palsy due to oxygen deprivation (OD), cerebral palsy related to neonatal icterus (NI), and neurological sequelae after TBI. In this study, we aimed to identify the concentrations of mononuclear cells (MNCs) and CD34+ cells in the bone marrow of these patients and their correlation with treatment outcomes.

## Methods

### Patients

All procedures were carried out in accordance with the local and national regulatory guidelines. The procedures followed the ethical standards described by the Helsinki Declaration. Eighty-eight bone marrow samples were included in the study after obtaining ethics committee approval from Vinmec International Hospital and after proper informed consent.

The age of the patients ranged from 2 to 50 years. The samples were grouped into two categories based on age: 2 to 16 years—Group I (patients with CP), 20–50 years—Group II (patients with neurological sequelae after TBI). The time from disease onset to stem cell application ranged from 1 to 36 months. The exclusion criteria were coagulation disorders, severe health conditions such as cancer, failure of the heart, lungs, liver, kidneys, or any active infection.

### Bone marrow aspiration and cell isolation

Bone marrow aspiration was conducted from the patients’ bilateral anterior iliac crest under general anesthesia in an operating theater at Vinmec Times City International Hospital. The required bone marrow volume was calculated by each participant’s body weight. Based on our prior experience, this volume was determined as follows: 8 mL/kg for patients who weighed less than 10 kg and [80 mL + [body weight in kg - 10] × 7 mL] for patients who weighed more than 10 kg, with a total volume of no more than 350 mL. BMMNC separation was performed using density gradient centrifugation with Ficoll and then infused intrathecally. The same procedure was repeated 6 months later. CD34+ hematopoietic progenitor cells were counted by flow cytometry using Stem-Kit Reagent from Beckman Coulter, and BMMNCs were stained with 7AAD, CD45-FITC, and CD34-PE, run in a Navios flow cytometer and analysed by Navios software.

### Clinical assessment

The assessment method for neurological sequelae after TBI and CP treatment was previously published. Gross motor function 88 items (GMFM-88) were used to evaluate the improvement in CP [22], and functional independence measure (FIM) scores were used to assess the changes in neurological sequelae after TBI [23].

### Statistical analysis

Descriptive statistics were used to illustrate the demographics of patients with diseases related to neurological conditions. Categorical variables were expressed in proportion, while quantitative variables were described by the mean value and standard deviation. A paired t test was used to determine the relationship between the percentage of CD34+ cells and sex, while one-way ANOVA was used to assess the difference between the percentage of CD34+ cells and age groups. The correlation between MNCs per mL BMMNC, percentage CD34+ and outcomes (GMFM-88, FIM) of diseases at baseline and after 6 months of infusion was assessed using multiple linear regression. A *p* value less than 0.05 was considered the threshold for significance. Data analyses were performed using STATA software version 14.0.

## Results

We collected data from 88 patients with three neurologic conditions (40 CP due to OD, 23 CP related to NI, and 25 neurological sequelae after TBI). Among these patients, CP due to OD and CP related to NI ranged in those aged under 17 years, whereas neurological sequelae after TBI ranged in those aged 20–50 years. Males accounted for 73.86% (65/88) of the patient cohort.

The density of MNCs per mL in patients with three neurologic conditions contained 5.22 ± 4.24 million cells, and the percentage of CD34+ cells accounted for 5.03 ± 4.42%. The average number of MNCs per mL differed significantly by type of disease, with the highest in patients with CP due to OD. There was a significant difference in the percentage of CD34+ cells by the type of disease, with the highest in CP related to NI. The patients aged under 17 years had an average number of MNCs per mL of 6.21 ± 4.64 million cells, which was significantly higher than that in those aged 20–50, with 2.73 ± 0.68 million cells (*p* value = 0.0004). There was a significant difference in the percentage of CD34+ cells by age group, with 6.42 ± 4.52% for patients aged under 17 years and 1.54 ± 0.50% for those aged 20–50 years (*p* value < 0.0001). The results also showed that there was no significant difference between the number of MNCs per mL and % CD34+ by gender (*p* value > 0.05) (please see details in Table 1).

**Table 1:** The average MNCs per mL and the percentage of CD34+ cells separated from bone marrow in three neurologic conditions

	MNCs per mL (10^**6**^)	Percentage of CD34+ cells
	mean ± SD	mean ± SD
Age (year)		
*< 17*	6.21 ± 4.64	6.42 ± 4.52
*20–50*	2.73 ± 0.68	1.54 ± 0.50
	*p* = 0.0004	*p* < 0.0001
Gender		
*Male*	5.09 ± 4.25	5.02 ± 4.65
*Female*	5.57 ± 4.27	5.05 ± 3.79
	*p* = 0.6432	*p* = 0.9809
Type of disease		
*CP due to OD*	7.91 ± 4.85	4.59 ± 1.57
*CP related to NI*	3.24 ± 2.12	9.58 ± 6.06
*Neurological sequelae after TBI*	2.73 ± 0.67	1.54 ± 0.50
	*p* < 0.0001	*p* < 0.0001
**All**	**5.22 ± 4.24**	**5.03 ± 4.42**

**Fig. 1:**
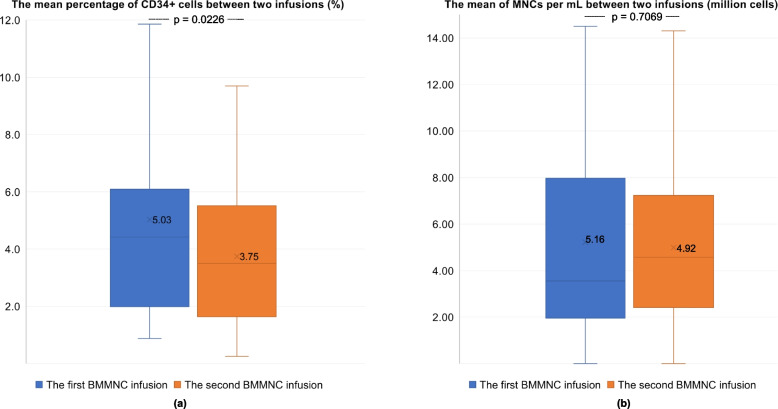
The mean percentage of CD34+ cells and the number of MNCs per mL between the two infusions: **a** The percentage of CD34+ cells; **b** the average density of MNCs

The mean percentage of CD34+ cells in the first infusion was 5.03%, which was significantly higher than that in the second infusion at 3.74% (*p* value = 0.0226). There was no difference between the number of MNCs per mL between the two infusions (*p* value = 0.7069 > 0.05) (please see details in Fig. 1).

**Fig. 2:**
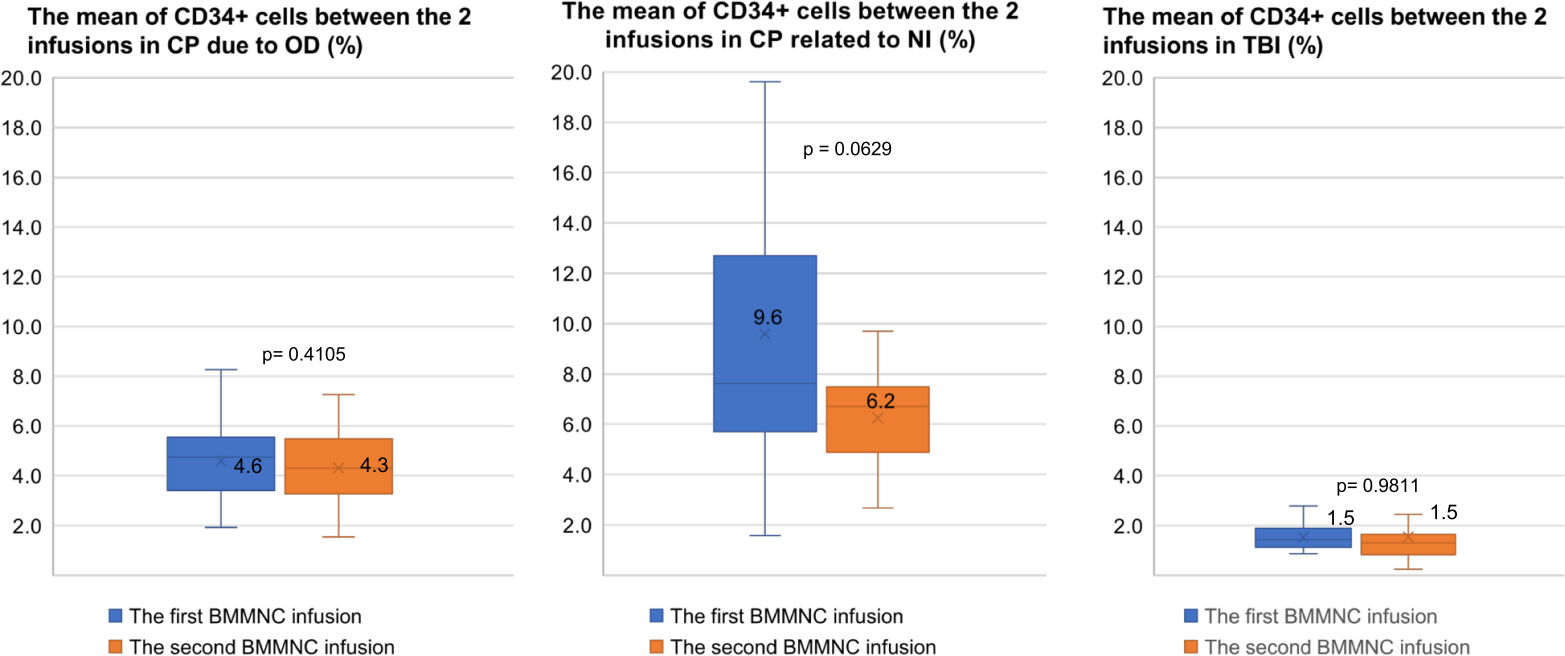
The mean percentage of CD34+ cells between the two infusions by diseases

**Fig. 3:**
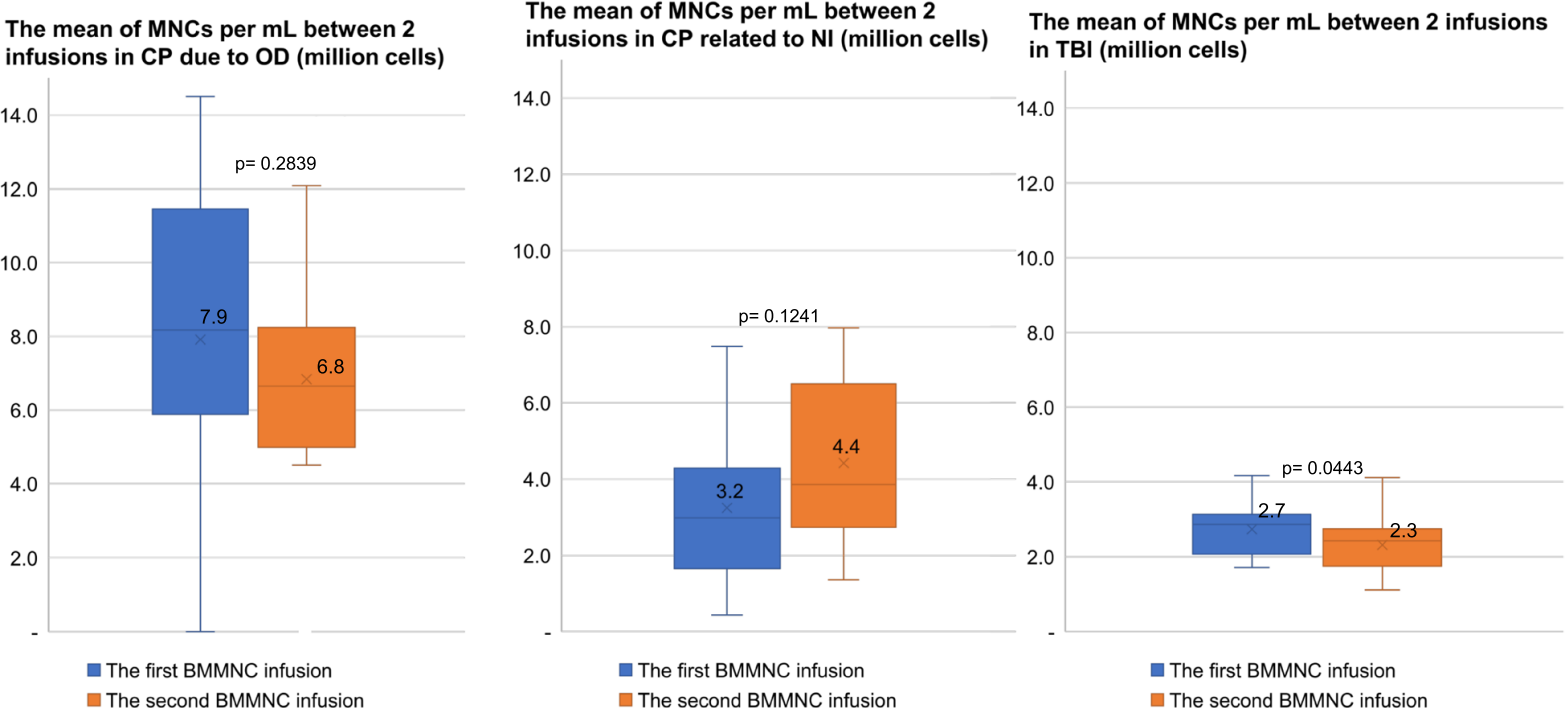
The mean number of MNCs per mL between the two infusions by diseases

There was no significant difference between the mean percentage of CD34+ cells between the two infusions by disease (*p* value > 0.05) (Fig. 2). However, the number of MNCs per mL in patients with neurological sequelae after TBI in the first infusion was 2.7 million cells, which was significantly higher than the figure in the second infusion at 2.3 million cells (*p* value = 0.0443) (Fig. 3).

**Fig. 4:**
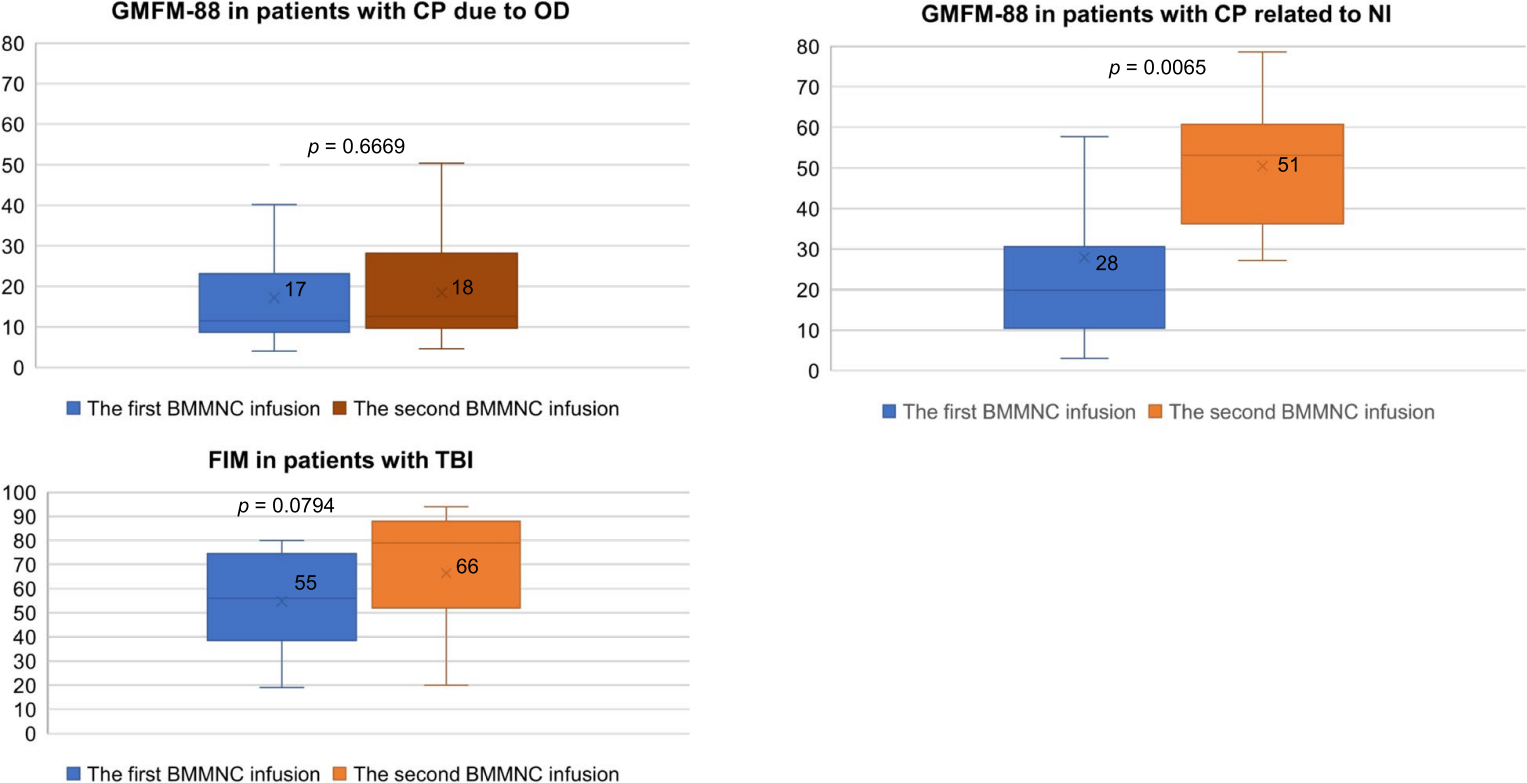
The improvement of outcomes in patients with neurologic conditions over-time: gross motor function (GMFM-88) in patients with CP due to OD, CP related to NI; Functional independence measure (FIM) in patients with neurological sequelae after TBI

Gross motor function (GMFM-88) in patients with CP related to NI improved significantly over-time (*p* value = 0.0065) (Fig. 4).

**Fig. 5:**
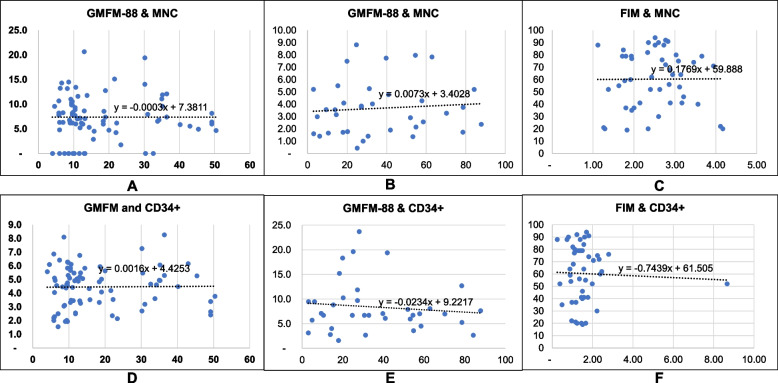
The correlation between the outcome of BMMNC infusion for CP due to OD, CP related to NI, and neurological sequelae after TBI with the concentration of MNCs and the percentage of CD34+: **A** and **D** correlation between MNC, CD34+ in patients with CP due to OD; **B** + **E** correlation between MNC, CD34+ in patients with CP related to NI; **C** + F correlation between MNC, CD34+ in patients with neurological sequelae after TBI

There was no correlation between the number of MNCs and the percentage of CD34+ cells with the outcomes of BMMNC infusion in either CP, or TBI (*p*-value> 0.05) (Fig. 5).

The multivariable regression model showed the percentage of CD34+ association with the outcome (GMFM-88) in patients with CP (OD, NI). Overall, the data implied that an increase in CD34+ percent led to a decrease in GMFM-88 by 3.64 points (*p* value = 0.001). There was no correlation between the density of MNCs and the percentage of CD34+ cells or the FIM score in patients with neurological sequelae after TBI (*p* value > 0.05) (please see details in Table 2).

**Table 2:** The relationship between MNCs per mL and the percentage of CD34+ by the outcomes of diseases after 6 months (GMFM-88 for CP; FIM for neurological sequelae after TBI)

Variables	GMFM-88		FIM	
	*Beta*	*95% CI*	*Beta*	*95% CI*
% CD34+	−3.64*	[−5.62, − 1.66]	−0.50	[−2.50, 1.51]
MNCs per mL	−0.36	[−0.90, 0.27]	− 0.70	[−6.19, 4.80]
	*F*(2,50) = 7.92, *p* = 0.001, *R*^2^ = 0.241		*F*(2,20) = 0.27, *p* = 0.7655, *R*^2^ = 0.0264	

*p value < 0.05

## Discussion

In recent years, stem cell therapy for different neurologic conditions has become a new hope for patients. Bone marrow mononuclear cell therapy was one of the initial cell types used in clinical trials for the treatment of cerebral palsy [4, 6, 10, 24–27] and TBI [8, 20]. Aging, injury, and acquired diseases can significantly change BMMNC components [28]. Some of our clinical trials using autologous BMMNCs for CP have shown the safety and initial effectiveness of the therapy [7, 10, 27, 29, 30].

Our results indicated that the average MNC per ml and percentage of CD34+ cells in CP patients (aged under 17) were significantly higher than those in patients with neurological sequelae after TBI (aged 20 and over) (*p* value < 0.05). In patients aged under 17, the average MNC per ml and percentage of CD34+ cells in our study were 6.21 million cells and 6.42%, respectively, which were higher than those in spinal cord injury patients, which were 4.71 million cells and 1.05%, respectively. In contrast, the average number of MNCs per ml in our study was 2.73 million cells, lower than that of patients with spinal cord injury, 4.03 million cells in the same aged 20–50 [28].

Our findings show that the average density of MNCs in patients with neurologic conditions with 5.2 million cells, was lower than the average of 7.5 million cells in a healthy person [31]. In our study, the figures for children with CP related to NI and CP due to OD were 7.9 million cells, and 3.2 million cells, respectively. These values were similar to those in children with spinal cord injury (4.7 million cells) [28], and children with CP (10.2 million cells) [26] but higher than those in children with traumatic brain injury (2.1 million cells) [32], liver cirrhosis (1.5 million cells) [33], patients with critical limb ischemia (3.2 million cells) [34] and patients with spinal cord injury (3.9 million cells) [28] (Table 3).

**Table 3:** The density of BMNC in patients with different diseases

No.	Author	Sample size	Patients/Donors	Mean quantity of MNCs/mL (10^**6**^)
1	Ema (1990) [35]	12	Donors	NA
2	Chernykh (2006) [31]	10	Heatlhy adults	7.5
4	Mohamadnejad (2007) [33]	4	Liver cirrhosis	1.5
5	P Hernández (2007) [34]	12	limb ischaemia	3.2
7	Dedeepiya (2012) [28]	332	Spinal Cord Injury	3.9

The average percentage of CD34+ cells was 5.03%, higher than that in healthy adults (2%) [35–41]. There was a difference between the types of disease (*p value = 0.0006*), with the highest average percentage of CD34+ cells in CP related to NI (9.6%), followed by CP due to OD (4.6%), and neurological sequelae after TBI (1.5%). Since limited publications report on the percentage of bone marrow CD34+ cells in children with neurologic conditions, we could only compare this value in our current study to studies in spinal cord injury patients (in a 0–20 years old group), i.e. a study by Chernykh et al. (reporting 5.4%) [31], and Deep et al. (reporting 1.05%) [28], children with spinal trauma (1.05%) [28], spinal cord injury (5.4%) [31], and children with traumatic brain injury (2.1%) [32]. Nevertheless, the percentage of CD34+ cells in children with CP related to NI in our current study was the highest. Based on our current study, these comparisons suggest a decrease in the density of MNCs and an increase in the percentage of CD34+ cells in children with neurologic conditions.

Gross motor function (GMFM-88) in both children with CP due to OD and CP related to NI [27] improved remarkably over-time. Similar outcomes were observed in other clinical trial studies that applied cell therapy combined with rehabilitation in the treatment of cerebral palsy [42–45]. These results show that patients who received stem cell therapy exhibited a significant improvement compared with those who only received rehabilitation. In addition, age ≥ 36 months is associated with poor gross motor function [46]. These findings should be confirmed in larger, multicenter, placebo-controlled trials.

The functional independence measure (FIM) effectively evaluates a patient’s functional abilities extensively covering cognitive and motor domains. In our study, FIM in patients with neurological sequelae after TBI improved over time. This level of improvement was observed in other studies that applied cell therapy in the treatment of TBI [9, 19, 47]. In addition, the age group of < 18 years in patients with TBI showed improvement compared to patients in the age group of > 18 years [9].

Interestingly, in the current study, when analysing the correlation of MNCs and CD34+ cell density with the outcome of patients with CP 6 months after autologous BMMNC infusion, we found that the change in the percentage of CD34+ cells correlated with the improvement in GMFM-88 values. An increase in CD34+ cells per percent led to a decrease in GMFM-88 scores by 3.64 points (*p* value = 0.001). This suggests that treatment outcomes could depend on the density of CD34+ cells. In the present study, we found that there was no correlation between the percentage of CD34+ cells and the FIM score at baseline or after 6 months.

## Conclusions

In conclusion, the density of bone marrow mononuclear cells was 5.22 million cells per mL and 5.03% of cells were CD34+ cells in patients with different neurologic conditions. The average MNC per ml and the percentage of CD34+ cells in the bone marrow of CP patients (aged under 17) were higher than those in patients with neurological sequelae after TBI (aged 20 and over). The highest density of MNCs in each ml of bone marrow was found in patients with CP due to OD, whereas the percentage of CD34+ cells was the highest among patients with CP related to NI.

## Supplementary Information

**Additional file 1.** The dataset file of study on autologous bone marrow mononuclear cells for patients with cerebral palsy and neurological sequelae after traumatic brain injury.

## Data Availability

All data generated or analysed during this study are included in this published article and its supplementary file.

## Abbreviations

BMMNC: Bone marrow mononuclear cell
CP: Cerebral palsy
FIM: Functional independence measure
MNCs: Mononuclear cells
NI: Neonatal icterus
OD: Oxygen deprivation
TBI: Traumatic brain injury
